# Amino acid digestibility and metabolizable energy of soybean meal of different origins in cecectomized laying hens^1^

**DOI:** 10.1016/j.psj.2023.102580

**Published:** 2023-02-14

**Authors:** W. Siegert, S. Kuenz, W. Windisch, M. Rodehutscord

**Affiliations:** ⁎ Institute of Animal Science, University of Hohenheim, 70599 Stuttgart, Germany; † TUM School of Life Sciences Weihenstephan, Technical University of Munich, 85354 Freising, Germany

**Keywords:** soybean meal, laying hen, amino acid digestibility, metabolizable energy

## Abstract

This study investigated the variation in amino acid (**AA**) digestibility and MEn of 18 samples of solvent-extracted soybean meal (**SBM**; 6 European, 7 Brazilian, 2 Argentinian, 2 North American, 1 Indian) in cecectomized laying hens. The experimental diets contained either 300 g/kg of cornstarch or one of the SBM samples. Pelleted diets were fed to 10 hens in two 5 × 10 row-column designs so that 5 replicates were obtained from each diet during 5 periods. A regression approach and the difference method were used to determine AA digestibility and MEn, respectively. The variation in the digestibility of SBM differed among AA with ranges in digestibility of 6 to 12%-units for most AA. Among the first-limiting AA, the digestibility was 87 to 93%, 63 to 86%, 85 to 92%, 79 to 89%, and 84 to 95% for Met, Cys, Lys, Thr, and Val, respectively. The range of MEn for the SBM samples was 7.5 to 10.5 MJ/kg DM. Indicators of SBM quality (including trypsin inhibitor activity, KOH solubility, urease activity, and in vitro N solubility) and analyzed SBM constituents were significantly correlated (*P* ≤ 0.05) with AA digestibility or MEn only in a few cases. No differences were observed in AA digestibility and MEn between countries of origins, except low digestibility of some AA and MEn for the 2 Argentinian SBM samples. These results suggest that the precision of feed formulation benefits from considering the variations in AA digestibility and MEn. Often used indicators for SBM quality and analyzed constituents were not suitable to explain variations in AA digestibility and MEn, suggesting that AA digestibility and MEn are determined by other factors.

## INTRODUCTION

Knowledge of the contribution of feed ingredients to meet the amino acid (**AA**) and energy requirements of animals is needed for precise feed formulation. Imbalanced AA supply to the animals increases N excretion by the animals. Excretion of N is associated with negative effects, including N leakage into groundwater, eutrophication, and health issues caused by ammonia (Siegert and Rodehutscord, 2019; Cappelaere et al., 2021). There is common consensus among nutritionists that the AA requirement of poultry and concentrations of the feed are best expressed on a digestible AA basis.

Digestibility determination should exclude postileal fermentation because it affects the AA composition of digesta (Thornburn and Willcox, 1965) and postileal AA absorption is quantitatively not relevant (Webb, 1990). For adult birds, such as laying hens, determination of AA digestibility using total excreta of cecectomized birds has the advantage that the major location of microbial fermentation in birds is removed (Parsons, 1984). Total excreta collection allows to conduct experiments with only a few animals, and cecectomized laying hens can be used over a long period of time; AA digestibility was shown to be constant over the age period of 32 to 76 wk (Zuber, 2017). In addition, large sample sizes based on total excreta collection are obtained from the same animals, reducing sources of error of AA digestibility determination compared with the spot-sampled digesta from the terminal ileum of slaughtered birds (Rezvani et al., 2008).

Processed soybeans, especially solvent-extracted soybean meal (**SBM**), are commonly used protein-rich ingredients in feed for poultry worldwide. Feeding soybeans is criticized because of deforestation in some soybean-producing areas and nutrient accumulation in areas where imported soybeans are fed to animals (Da Silva et al., 2010). However, soybean protein has higher concentrations of limiting AA than most other proteins, such as those of other oilseed meals, peas, lupins, and faba beans (Feedipedia, 2022). Furthermore, AA digestibility of SBM was shown to be higher than that of other oilseed meals or legumes in broiler chickens (Bryden and Li, 2010; Blok and Dekker, 2017).

Despite the important role of SBM in common laying hen feed, information on the variability of AA digestibility and MEn of SBM in laying hens is scarce. Adedokun et al. (2014) found small differences in AA digestibility of 3 SBM samples sourced from the United States. To our knowledge, other studies investigating variation in AA digestibility of SBM in laying hens are not available. In broiler chickens, comprehensive studies found larger variations in AA digestibility of 6 (de Coca-Sinova et al., 2008), 22 (Frikha et al., 2012), and 55 (Ravindran et al., 2014) SBM samples sourced from Argentina, Brazil, the United States, and India. For MEn, studies examining variations in laying hens are not available, but studies on broiler chickens found a considerable variation in MEn or ME in SBM (Ravindran et al., 2014; Leung and Kiarie, 2020). These differences were discussed by the authors to be caused by cultivars, cultivation conditions, processing methods, and antinutritive substances. The variations in AA digestibility and MEn of SBM in broiler chickens suggest that a considerable variation may also exist in laying hens. However, data on AA digestibility and MEn determined in one type of chicken should not be applied in another type (Adedokun et al., 2009).

The objective of this study was to examine the variations in AA digestibility excluding basal endogenous AA losses and MEn concentrations among 18 SBM variants of different origins in cecectomized laying hens. We also investigated relationships between various analytical fractions of SBM and AA digestibility or MEn aiming to explain the variations.

## MATERIALS AND METHODS

### Soybean Meal Variants

Eighteen SBM variants were investigated. Six of the SBM variants were of European, 7 were of Brazilian, 2 were of Argentinian, 2 were of North American, and 1 was of Indian origin (see Supplementary Table 1 for detailed description of origins). All the European and 2 Brazilian variants were not genetically modified (**non-GMO**), while the remaining variants were genetically modified (**GMO**). The concentration ranges of CP and the first-limiting AA Met, Cys, Lys, and Thr in the SBM variants were 498 to 575 g/kg DM, 7.0 to 8.9 g/kg DM (1.4–1.6 g/16 g N), 6.6 to 8.2 g/kg DM (1.3–1.5 g/16 g N), 30.1 to 39.7 g/kg DM (5.8–7.2 g/16 g N), and 19.0 to 25.3 g/kg DM (3.9–4.6 g/16 g N), respectively (Table 1; Supplementary Table 1). The average particle size ranged from 0.45 mm to 1.30 mm. Some SBM variants contained large, solid particle agglomerates, which is reflected in the measured particle sizes of >4.0 mm.

**Table 1:** Analyzed concentrations of soybean meal variants (g/kg DM, unless otherwise stated).

Item	Median	SD	Minimum	Maximum	CV (%)
DM (g/kg)	890	6.5	883	912	0.7
CP	519	23.5	485	564	4.5
Crude fat	22	4.6	15	32	20
Crude ash	75	7.2	70	100	9.3
Crude fiber	44	8.9	30	71	20
aNDF_om_^1^	164	34	110	232	21
ADF_om_^2^	69	13	55	102	18
NDF-N^3^	6.2	4.2	3.1	20.3	56
ADF-N^4^	1.0	0.2	0.7	1.6	23
Starch	56	8	46	70	13
Sugar	11	1.4	8	13	13
Gross energy (MJ/kg DM)	20.9	0.6	19.5	21.2	2.9
Amino acids (g/16 g N)					
Ala	4.6	0.18	4.3	5.2	3.9
Arg	7.2	0.41	6.9	8.5	5.6
Asx	12.0	0.50	11.7	13.8	4.1
Cys	1.4	0.05	1.3	1.5	3.4
Glx	19.7	0.85	18.3	21.5	4.4
Gly	4.4	0.17	4.2	5.0	3.9
His	2.9	0.13	2.7	3.3	4.3
Ile	5.0	0.25	4.6	5.5	5.0
Leu	8.1	0.33	7.9	9.3	4.0
Lys	6.4	0.29	5.8	7.2	4.6
Met	1.5	0.05	1.4	1.6	3.7
Phe	5.6	0.37	5.1	6.3	6.6
Pro	5.2	0.22	4.9	6.0	4.2
Ser	5.2	0.29	5.0	6.2	5.5
Thr	4.2	0.18	3.9	4.6	4.3
Tyr	3.5	0.15	3.3	4.0	4.2
Val	4.9	0.22	4.6	5.6	4.4
Tannins					
Total phenols	0.25	0.04	0.17	0.36	16
Tannin phenols	0.27	0.04	0.19	0.36	14
Condensed tannins	<0.05^5^				
Protein dispersibility index (%)	13	4.0	6	20	31
KOH solubility (%)	60	7.9	36	71	14
Trypsin inhibitor activity	2.9	1.1	0.8	4.5	40
Urease activity (mg N/g/min)	<0.05^6^				
In vitro N solubility (%)	94	1.1	92	96	1.2
InsP_6_^6^	21.9	1.9	18.6	25.5	8.8
Particle size distribution^7^					
Mean	0.93	0.20	0.45	1.30	22
Heterogeneity	3.1	1.7	2.2	9.8	49

Values of individual soybean meal variants are presented in Supplementary Table 1.

1 Neutral detergent fiber, determined without residual ash and after treatment with α-amylase.

2 Acid detergent fiber, determined without residual ash.

3 Neutral detergent insoluble nitrogen.

4 Acid detergent insoluble nitrogen.

5 Below the limit of quantification for all SBM samples.

6 Concentrations of partially dephosphorylated inositol phosphate isomers are described in Supplementary Table 1.

7 Means values and heterogeneity determined as the parameter estimates described in Eq. (4).

### Diets

The experimental diets contained either 300 g cornstarch/kg or 300 g/kg of one of the SBM variants (Table 2) so that SBM as the test ingredient was included in one inclusion level, as suggested by an evaluation of the assay (Siegert, 2022). The diet containing cornstarch was formulated to comply with the recommendations of the Gesellschaft für Ernährungsphysiologie (1999) for laying hens weighing 1,800 g and a daily egg production of 60 g. All ingredients, except cornstarch and the SBM variants, were mixed. Thereafter, this basal mixture was divided in equal portions, which were complemented with cornstarch or one of the SBM variants. The SBM variants were not ground prior to mixing. The complete diets were pelleted through a 3-mm die without using steam. Analyzed nutrient concentrations in the experimental diets (Supplementary Table 2) confirmed the calculated values.

**Table 2:** Composition of the experimental diets (g/kg).

Feed ingredients	Basal diet	Diets containing soybean meal variants
Corn		322
Soybean meal		135
Wheat gluten feed		90
Limestone		60
Grass meal		40
Soybean oil		30
Premix^1^		20
l-Lys·sulfate		2.5
dl-Met		0.5
Cornstarch	300	-
Soybean meal variant (no. 1–18)	-	300

1 Provided per kg of diet: 10.2 g calcium carbonate, 4 g calcium sodium phosphate, 2.4 g monocalcium phosphate, 1.4 g sodium chloride, 0.6 g sodium carbonate, 10,000 IE vitamin A (retinylacetate), 2,000 IU vitamin D3 (cholecalciferol), 20 mg vitamin E (all-rac-α-tocopheryl acetate), 3 mg vitamin K3 (menadione sodium bisulfite), 1.6 mg vitamin B1 (thiamine mononitrate), 4 mg vitamin B2 (riboflavin), 2.4 mg vitamin B6 (pyridoxine hydrochloride), 16 μg vitamin B12 (cyanocobalamin), 20 mg niacinamide, 6 mg calcium-d-pantothenate, 0.6 mg folic acid, 0.2 mg biotin, 250 mg choline chloride, 60 mg iron from iron-(II)-sulfate monohydrate, 60 mg manganese from manganese-(II)-sulfate monohydrate, 32 mg zinc from zinc-oxide, 16 mg zinc from zinc-sulfate monohydrate, 4 mg copper from cupric-(II)-sulfate pentahydrate, 0.6 mg iodine from calcium iodate anhydrous, 0.2 mg selenium from sodium selenite.

### Animals and Housing

Laying hens of the strain LSL-Classic were cecectomized at 23 wk of age following the procedures described in detail by Zuber et al. (2016a) with modifications by Siegert et al. (2022a). The same hens were used for another AA digestibility study (Siegert et al., 2022a) prior to the present study. All procedures were conducted in accordance with the German animal welfare regulations and were approved by the Regierungspräsidium Stuttgart under protocol no. V352/18TE. The age range of the birds during the study was 55 to 80 wk with the experiment being interrupted for several wk due to COVID-19 restrictions. Bird health status was monitored at least once daily. During this study, the hens were kept individually in metabolism units, measuring 89 cm × 89 cm × 89 cm, equipped with perches, nest boxes, feeding troughs, water cups, and a wire mesh floor. Stainless steel trays were installed under the mesh for excreta collection. When not housed in the metabolism units, the cecectomized laying hens were in groups together with accompanying hens in a floor pen of 2.2 m × 7.0 m with straw and wood shavings as litter material. This pen was equipped with wooden perches, feeding and water troughs, nest boxes, and sand baths. The temperature was set to 20°C and lighting was switched on from 07:00 h to 21:00 h.

### Experimental Design and Procedures

Two resolvable 5 × 10 row-column designs were used to test 5 replicates of each of the experimental diets using 10 hens during 5 periods. The first row-column design comprised the basal diet and the diets containing SBM variants no. 1, 2, 4, 5, 6, 7, 9, and 10, with the other diets being assigned to the second row-column design. The design was optimized using the OPTEX procedure of the SAS software package (Version 9.4, SAS Institute, Cary, NC). In some cases, eggs cracked, and observations could not be included because the egg content mixed with excreta. The missing observations were obtained during an 11th period.

The hens received the respective diet for 4 d of adaptation during each period. Then, beginning at 07:00 h and 15:00 h on each day, excreta were completely collected for 4 d and immediately frozen after collection. Spilled pellets, feathers, and shed skin flakes were removed from the trays prior to each collection. Collected excreta were later pooled so that 1 hen in each period represented 1 observation. While housed in the metabolism units, 115 g of feed was provided daily, offered in equal meals at 07:00 h and 15:00 h. Feed residues in feed and water troughs were collected, frozen for storage, and later dried at 105°C to correct feed intake on a DM basis. Egg weight was recorded daily. Hens were moved into the floor pen for at least 2 d after each period, where a standard layer feed was provided for ad libitum consumption.

### Sample Preparation and Chemical Analyses

A centrifugal mill (ZM 200, Retsch GmbH, Haan, Germany) equipped with a 1.0-mm sieve was used to grind SBM variants and experimental diets for analysis of crude fiber, neutral detergent fiber determined without residual ash and after treatment with α-amylase (**aNDF_om_**), and acid detergent fiber determined without residual ash (**ADF_om_**). Other crude nutrients were analyzed after grinding through a 0.5-mm sieve using the same mill. All other analyses of SBM variants and experimental diets were conducted after grinding to a fine powder using a ball mill (MM40, Retsch GmbH, Haan, Germany). Excreta samples were thawed at 4°C, weighed, homogenized with a hand blender, and the DM content was determined in triplicates. The remaining excreta were freeze-dried and then pulverized with a vibrating cup mill (Pulverisette 2, Fritsch GmbH, Idar-Oberstein, Germany).

The official methods for nutrient analyses in Germany (Verband Deutscher Landwirtschaftlicher Untersuchungs- und Forschungsanstalten, 2007) were used to analyze the DM (no. 3.1), CP (no. 4.1.1), crude fat (no. 5.1.1b), crude ash (no. 8.1), crude fiber (no. 6.1.1), starch (no. 7.2.1), sugar (7.1.4), aNDF_om_ (no. 6.5.1), ADF_om_ (no. 6.5.2), urease activity (no. 20.1), and the protein dispersibility index (no. 20.2). The KOH solubility and trypsin inhibitor activity (**TIA**) were analyzed according to the EN ISO 14244:2014-02 and EN ISO 14902:2001 standards, respectively. Neutral detergent insoluble N (**NDF-N**) and acid detergent insoluble N (**ADF-N**) were analyzed as described previously (Haese et al., 2022). The in vitro N solubility was determined according to Zuber et al. (2016a). Bomb calorimetry was used to determine gross energy. The AA were analyzed following Rodehutscord et al. (2004) with modifications (Siegert et al., 2017). Glycine was not analyzed in excreta because parts of the measured Gly may be formed from uric acid during sample hydrolysis (Dalgliesh and Neuberger, 1954). The amide residues of Asn and Gln are lost during acid hydrolysis, leading to the formation of Asp and Glu, respectively (Fontaine, 2003). Therefore, Asn and Gln were determined together with Asp and Glu and presented as Asx and Glx, respectively. Inositol phosphates were analyzed as described by Zeller et al. (2015), whereas tannins were analyzed as described by Wischer et al. (2013). The particle size distribution of the soybean variants was determined according to Krieg et al. (2021) using the sieve sizes 20, 10, 6.3, 4.0, 2.0, 1.18, 1.0, 0.5, 0.25, 0.125, and 0.063 mm.

### Calculations and Statistical Analysis

Amino acid digestibility of the SBM variants was determined by calculating the AA digestibility of the experimental diets (Supplementary Table 3) for each observation as the first step using the following equation:

(1) AAdigestibilityofdiets=(AAintake−AAexcreted)/AAintake

In the next step, AA digestibility of the SBM variants excluding basal endogenous losses was calculated using a regression approach (Rodehutscord et al., 2004). In this approach, AA digestibility of test ingredients represent the fitted linear regression between digested and ingested AA amounts. The fitted model used by Siegert et al. (2022a) to calculate and statistically compare AA digestibility of the SBM variants is shown below:

(2) $$y_{ijk}=\mu +\beta_{i}\times x_{ijk}+\omega \times z_{ijk}+h_{j}+p_{k}+e_{ijk}$$

where *y_ijk_* is the digested amount of the corresponding AA from hen *j* during period *k* fed with variant *i, µ* is the intercept, *β_i_* and *ω* are the variant-specific and the general slope of the digested amount on the consumed amount of the AA (*x_ijk_*) and feed intake (*z_ijk_*), respectively, h*_j_* and p*_k_* are the random effects of hen *j* and period *k*, respectively, and e*_ijk_* is the residual error. The AA concentration of the basal diet was subtracted from the concentration of diets prior to analysis to achieve *x_ijk_* = 0 for the basal diet. The data were graphically checked for normal distribution and homogeneous variance of residuals. The statistical evaluation was conducted using the MIXED procedure of SAS. Additionally, a toasted soybean sample was included in the first row-column design. This sample was included in the statistical evaluation because of its contribution to the random hen and period effects.

The MEn of the SBM variants was determined according to Siegert et al. (2022a) by first calculating the MEn of the experimental diets for each observation using the following equation:

(3) $$\text{MEn}\;=\;\frac{a\;-\;b\;-\;36.5\;\text{kJ}\;/\;g\;\times \;(c\;-\;d\;+\;e)}{f}$$

where a is the gross energy intake (kJ/d), b is the gross energy excretion via the excreta (kJ/d), c is the N intake (g/d), d is the N excretion via the excreta (g/d), e is the N accretion in eggs (g/d), and f is feed intake (g DM/d). The MEn of the SBM variants was determined using the difference method according to the following equation:

(4) $$\text{MEn}\;(\text{MJ}\;/\;\text{kg}\;\text{DM})\;=\;\frac{g\;-\;h\;+\;i\;\times \;j}{j}$$

where g is the MEn of the diet containing the SBM variants (MJ/kg DM), h is the MEn of the basal diet (MJ/kg DM), c is the MEn of cornstarch (MJ/kg DM), and j is the proportion of the SBM variants in the diets. Feed intake was dropped as a covariate and MEn concentrations were used instead of ingested and digested amounts in Eq. (2) to evaluate MEn.

Particle size distribution of the SBM variants was evaluated following the procedures of Siegert et al. (2018) using the following regression:

(5) $$y=100/\left[1+e^{\left(-a\times (x-b)\right)}\right]$$

where y is the cumulative proportion of particles smaller than the sieve size x, a is the rate constant of the regression, and b is the inflection point. These regressions were computed using the NLMIXED procedure of SAS. Descriptive statistics and correlations were computed using the MEANS and CORR procedures of SAS, respectively. Figures were created using GraphPad Prism 8 (GraphPad Software Inc., San Diego, CA).

## RESULTS

### Hen Performance

Feed intake was 114 g DM/d (SD 2 g DM/d) and complete or close to complete in most observations. Hen and egg weights averaged 1,692 g (SD 120 g) and 64.4 g (SD 5.5 g) and decreased slightly and increased during the experiment, respectively. Out of the 8 d of each period, the hens laid eggs on at least 7 d in most observations. All hens laid eggs on at least 3 of the 4 d of excreta collection throughout the experiment. The average laying rate in the periods was 96%.

### Soybean Meal Variants

The medians of digestibility of the first-limiting AA in the SBM variants were 91% for Met, 78% for Cys, 90% for Lys, 84% for Thr, and 88% for Val (Tables 3 and 4). The AA with the highest digestibility (94%) and the lowest range in digestibility (3.5%-units) was Arg, while the AA with the lowest digestibility and the highest range in digestibility (23%-units) was Cys. The range in AA digestibility of all other SBM variants was between 6 and 12%-units. The median of MEn of the SBM variants was 9.5 MJ/kg DM with a range of 7.5 to 10.5 MJ/kg DM (Table 5). The variability in MEn among the SBM variants was higher (8% CV) compared to the variability in the digestibility of most AA (1–4% CV), except Cys with 8% CV).

**Table 3:** Digestibility of essential amino acids of the soybean meal variants (%).

Variant no.	Arg	His	Ile	Leu	Lys	Met	Phe	Thr	Val
1	93.0^cd^	88.0a–c	90.3b–d	86.9^bc^	90.4^a^	90.3^ab^	91.2b–e	84.1a–d	85.0^ef^
2	94.6a–c	89.0^ab^	91.1a–c	88.8^ab^	91.7^a^	91.3^ab^	91.3a–e	85.2a–c	89.1bcd
3	95.2^ab^	90.5^a^	92.9^a^	91.3^a^	91.5^a^	93.1^a^	93.2^a^	88.5^a^	92.3^ab^
4	93.6b–d	89.2^ab^	92.0^ab^	88.4^b^	91.0^a^	90.1^ab^	92.5a–c	85.8^ab^	87.9d–f
5	94.1a–d	88.1a–c	90.5a–c	88.6^ab^	90.4^a^	90.7^ab^	91.4a–e	85.0a–c	88.3c–f
6	92.7^cd^	85.2c–e	90.3b–d	86.8^bc^	86.9^bc^	90.8^ab^	90.8c–e	83.0b–d	84.8^ef^
7	95.0a–c	87.8a–c	89.5b–d	87.5^bc^	89.5^ab^	92.2^a^	89.5^de^	84.9a–c	87.8d–f
8	92.2^d^	83.3^de^	87.1^d^	84.0^c^	84.7^c^	87.1^b^	86.2^g^	78.8^d^	85.0^ef^
9	95.0a–c	88.6a–c	90.1b–d	87.7^bc^	89.7^ab^	90.0^ab^	90.4c–e	83.2a–d	95.5^a^
10	93.9b–d	87.1a–c	89.7b–d	87.3^bc^	90.1^ab^	91.4^ab^	88.9e–g	83.2a–d	87.2d–f
11	94.0b–d	85.3c–e	89.4b–d	87.9^b^	87.5^bc^	91.4^ab^	89.8^de^	82.4b–d	89.0b–e
12	94.8a–c	88.6a–c	90.8a–c	88.6^ab^	90.8^a^	90.9^ab^	90.3c–e	86.5^ab^	91.8a–c
13	95.7^a^	88.8^ab^	91.7a–c	90.1^ab^	91.1^a^	92.9^a^	91.8a–e	87.4^ab^	95.3^a^
14	92.9^cd^	82.6^e^	88.9^cd^	87.2^bc^	85.0^bc^	89.2^ab^	89.0^ef^	81.9b–d	86.9d–f
15	92.9^cd^	87.7a–c	88.6^cd^	85.5^bc^	90.2^ab^	90.2^ab^	87.7e–g	82.8b–d	84.5^ef^
16	92.2^d^	83.9c–e	87.3^d^	84.1^c^	87.0^bc^	87.2^b^	86.5^fg^	80.1^cd^	84.3^f^
17	93.8b–d	88.0a–c	92.1^ab^	89.2^ab^	91.1^a^	93.5^a^	92.9^ab^	86.7^ab^	87.7d–f
18	93.3^cd^	86.8b–d	91.1a–c	87.6^bc^	90.1^ab^	92.1^ab^	92.1a–d	84.3a–d	87.4d–f
Pooled SEM	0.9	2.2	1.6	1.8	2.2	1.9	2.0	2.5	3.4
Descriptive statistics									
Median	93.8	87.9	90.3	87.7	90.1	90.8	90.6	84.2	87.7
Range (%-units)	3.5	8.0	5.7	7.3	7.0	6.3	7.1	9.7	11.2
CV (%)	1	3	2	2	2	2	2	3	4

a–g Different superscript letters indicate significant differences between variants (*P* ≤ 0.050).

**Table 4:** Digestibility of nonessential amino acids of the soybean meal variants (%).

Variant no.	Ala	Asx	Cys	Glx	Pro	Ser	Tyr
1	80.2c–e	84.7a–d	73.9b–e	90.4^bc^	83.0^e^	84.5b–d	89.9e–g
2	84.2a–c	86.4^ab^	77.2a–d	91.2a–c	90.0^ab^	87.5a–d	92.3b–e
3	87.5^a^	88.5^a^	84.9^a^	93.2^a^	91.2^a^	90.8^a^	94.2a–c
4	84.1a–c	85.4a–c	78.3a–d	91.3a–c	87.6a–e	86.0b–d	91.7c–f
5	84.9^ab^	85.8a–c	77.9a–d	91.3a–c	88.5a–d	87.8a–d	91.6c–f
6	82.3b–e	81.3c–e	68.8^de^	89.3b–d	84.3^de^	83.3^cd^	89.0e–g
7	82.3b–e	86.2^ab^	81.4^ab^	89.5b–d	89.2a–c	88.1a–c	88.4^fg^
8	75.8^e^	80.4^de^	63.1^e^	84.5^ef^	84.9c–e	83.1^d^	86.2^g^
9	81.7b–e	84.6a–d	83.4^ab^	89.7b–d	87.7a–e	87.6a–d	97.3^a^
10	82.1b–e	86.1a–c	76.0a–e	89.4b–d	88.7a–d	87.4a–d	88.4^fg^
11	82.0b–e	82.2c–e	70.6c–e	87.2d–f	83.2^e^	86.5b–d	91.2c–f
12	83.6a–c	88.1^a^	82.5^ab^	90.8a–c	87.9a–e	89.3^ab^	93.1b–d
13	84.6a–c	87.1^a^	86.0^a^	91.4a–c	88.5a–d	89.3^ab^	94.8^ab^
14	82.1b–e	79.3^e^	74.1a–e	84.7^f^	86.4b–e	85.5b–d	88.9e–g
15	78.7c–e	86.1a–c	74.0b–d	87.5 ^–f^	88.2a–e	85.7b–d	85.5^g^
16	77.5^de^	82.9b–e	69.4^de^	86.6d–f	84.3^de^	83.7^cd^	86.3^g^
17	85.2^ab^	85.8a–c	80.6a–c	92.4^ab^	88.9a–d	87.5a–d	91.7c–f
18	83.1a–d	84.9a–c	78.0a–d	91.3a–c	87.3a–e	84.8b–d	91.8b–f
Pooled SEM	2.7	2.5	5.9	2.4	2.3	2.2	3.2
Descriptive statistics							
Median	82.3	85.6	77.6	90.1	87.8	86.9	91.4
Range (%-units)	11.7	9.2	22.8	8.6	8.3	7.6	11.7
CV (%)	3	3	8	3	3	3	3

a–g Different superscript letters indicate significant differences between variants (*P* ≤ 0.050).

**Table 5:** MEn of the soybean meal variants (MJ/kg DM).

Variant no.	MEn
1	9.0c–e
2	9.9a–c
3	9.7a–d
4	9.4b–d
5	10.0^ab^
6	10.5^a^
7	10.1^ab^
8	9.5a–d
9	9.9a–c
10	8.9^de^
11	9.2b–d
12	9.4b–d
13	8.9c–e
14	9.8a–d
15	9.9a–c
16	8.9^de^
17	8.0^ef^
18	7.5^f^
Pooled SEM	0.43
Descriptive statistics	
Median	9.5
Range (MJ MEn/kg DM)	3.0
CV (%)	8

a–f Different superscript letters indicate significant differences between variants (*P* ≤ 0.050).

Negative significant correlations (*P* ≤ 0.05) between AA digestibility and the analyzed aNDF_om_ concentration of SBM were observed for 4 AA (Asx, His, Lys, and Pro; r = −0.64 to −0.54) (Supplementary Table 4). Negative significant correlations (*P* ≤ 0.05) between AA digestibility and NDF-N and ADF-N were also observed for 4 AA (Asx, Glx, His, and Lys; r = −0.81 to −0.58 and r = −0.69 to −0.55, respectively), whereas correlations with sugar concentrations were significant (*P* ≤ 0.05) for 7 AA (Ala, Arg, Cys, Leu, Ser, Tyr, and Val; r = −0.57 to −0.48). Moreover, positive correlations (*P* ≤ 0.05) were observed between AA digestibility and KOH solubility for 4 AA (Asx, Glx, His, and Lys; r = 0.60–0.78), protein dispersibility index for 2 AA (Asx and Lys; r = 0.52 and r = 0.47, respectively), trypsin inhibitor activity for 4 AA (Asx, Glx, His, and Lys; r = 0.47–0.67), tannin phenols for 3 AA (Glx, His, and Lys; r = 0.49–0.51), and InsP_6_ concentration for 5 AA (Glx, His, Ile, Lys, and Phe; r = 0.50–0.59). Furthermore, negative correlations (*P* ≤ 0.05) between AA digestibility and in vitro N solubility of SBM were observed for 4 AA (Ala, Leu, Ser, and Tyr; r = −0.54 to −0.50). Correlations with AA concentrations were significant in some cases without any apparent pattern among the AA. Correlations with MEn were negative for crude fiber (r = −0.65) and positive for gross energy (r = 0.59).

## DISCUSSION

### Variation and Comparison With Literature

Considering the first-limiting AA in poultry feeds, variation in digestibility was larger for Cys, Thr, and Val (10–23%-units range) than for Met and Lys (6–7%-units range), indicating that using mean digestibility values in feed formulation implies different inaccuracies for different AA. Compared with the variation in AA digestibility of other common feed ingredients determined using the same assay, the variation in AA digestibility of SBM in the present study was intermediate. Summarizing the variation of the first-limiting AA Met, Cys, Lys, and Thr, similar or smaller ranges in digestibility were reported for rapeseed meal (n = 9, Rezvani et al., 2012), lupins, and peas (n = 12 each, Zuber et al., 2019). Overall, larger variations have been determined for faba beans (n = 16; Siegert et al., 2022a) and corn, rye, triticale, and wheat grains (n = 20 each) (Zuber et al., 2016a, b; Zuber and Rodehutscord, 2016, 2017).

Comparisons of AA digestibility of SBM with literature data determined using different animal assays are inexpedient because assay details markedly affect the results. Hence, comparisons made herein only refer to ranges and influencing factors where other assays were used. Compared with that in the present study, the range in AA digestibility of 3 SBM samples for laying hens investigated by Adedokun et al. (2014) was smaller (∼2%-units for most AA). The small number of SBM samples sourced from one harvest of one region of the United States might have contributed to the small variation determined by Adedokun et al. (2014). In broiler chickens, studies investigating AA digestibility of a larger number of SBM samples determined wider (∼20%-units, n = 55; Ravindran et al., 2014) and similar (∼5%-units, n = 22; Frikha et al., 2012) ranges in digestibility compared with those in the present study.

We are not aware of other studies investigating the variation in MEn of SBM in laying hens, and studies on variation in MEn of SBM in broiler chickens are scarce. Bertechini et al. (2018) determined 10.6 to 10.8 MJ MEn/kg DM of 3 Brazilian SBM samples in broiler chickens. This small variation was at the upper end of the MEn range determined in the present study using cecectomized laying hens. The variation in energy content of SBM in broiler chickens can be high, as shown by a span of 4.5 MJ ME/kg DM in a study of 55 SBM samples (Ravindran et al., 2014). However, Ravindran et al. (2014) did not correct ME to zero N accretion, which makes comparisons of the magnitude of energy content with the present study impossible. For our study, the determined MEn may be lower than that determined with noncecectomized laying hens because fermentation in the ceca can provide energy from prececally indigestible substrates.

The present study does not give any indication of systematic influences of the geographical origin on AA digestibility and MEn. The magnitude and variations in AA digestibility and MEn were similar among European, Brazilian, Argentinian, North American, and Indian variants (Supplementary Figure 1). The digestibility of several AA of the 2 Argentinian variants and the Indian variant was at the lower end of the variation compared with other origins. However, this may represent a coincident characteristic considering the low number of the Argentinean and Indian variants. In the literature, differences in AA digestibility among 3 and 22 SBM samples produced in 1 yr in different regions of the United States determined in laying hens (Adedokun et al., 2014) and pigs (Sotak-Peper et al., 2017), respectively, were small. No differences in AA digestibility among American (Argentina, Brazil, and the United States) SBM variants were found in broiler chickens, but lower AA digestibility was found in Indian SBM variants (Ravindran et al., 2014). In another study with broiler chickens, the digestibility of most AA was lower in Argentinian SBM variants compared with that in SBM variants from Brazil and the United States (Frikha et al., 2012). The ME of SBM also differed between geographical origins (Ravindran et al., 2014). These contradictions among studies may be attributable to year-dependent cropping conditions and processing techniques. The present study also does not give any indication of systematic differences in AA digestibility and MEn between non-GMO and GMO SBM variants (Supplementary Figure 2).

### Influences on Amino Acid Digestibility and MEn

We found no indication that TIA may have reduced AA digestibility because the few significant correlations between AA digestibility and TIA were positive. Correlations between TIA and AA digestibility of the complete diets were also positive for all and significant for 6 AA (Ala, Cys, Leu, Pro, Ser, and Val; *P* ≤ 0.038). Hence, there is no indication that the positive correlations between TIA and AA digestibility of the SBM variants are a methodological artifact of the separation of AA digestibility of the complete diets and that of the SBM variants. Positive correlations between TIA and AA digestibility of SBM were described previously in broiler chickens (Frikha et al., 2012; Ravindran et al., 2014). Contrarily, de Coca-Sinova et al. (2008) determined negative correlations between AA digestibility in broiler chickens and TIA in SBM. The TIA concentrations in our study and in the studies of Frikha et al. (2012) and Ravindran et al. (2014) were lower than in the study of de Coca-Sinova et al. (2008), and thus possibly too low to determine a negative relationship between AA digestibility and TIA. Some studies indicated that TIA concentrations in the range determined in our study (≤4.5 mg/g) can reduce AA digestibility in broiler chickens (Clarke and Wiseman, 2007; Kuenz et al., 2022). Perhaps, other characteristics of the SBM samples investigated in the present study masked AA digestibility reducing effects of low TIA concentrations, or the mature digestive tract of the hens compared to broiler chickens may have made TIA less relevant. Overall, the absence of relationships between AA digestibility and TIA suggest that the heat treatment was sufficient to avoid the diminishing effects of TIA on AA digestibility. Notably, consequences of TIA for AA digestibility probably were smaller than suggested by TIA concentrations in the SBM samples because of the heat input during pelleting of the diets. Heat treatment can also decrease AA digestibility when executed too extensively, for example, via Maillard reactions (Ravindran et al., 2014). The KOH solubility and the protein dispersibility index are often used as indicators of extensive heat treatment. There is no unambiguous indication of effects of extremely extensive heat treatment during SBM processing in our study. Nonetheless, the measured KOH solubility and protein dispersibility index values of several SBM variants were considerably below the often-recommended minimum values of ∼70 and ∼15%, respectively (Araba and Dale, 1990; van Eys and Ruiz, 2021). Despite the substantial shortfall of the recommended KOH solubility and protein dispersibility index values, correlations with digestibility were significant only for a few AA. This may indicate that KOH solubility and the protein dispersibility index are weak indicators of extremely extensive heat treatment of SBM. Possible reasons for inconsistencies in relationships between AA digestibility and the KOH solubility or the protein dispersibility index values among studies include the effects of particle size and cultivation conditions during soy cropping for measured protein solubility (summarized by Hemetsberger et al., 2021). Nonetheless, Maillard reactions caused by heat input may have been a relevant contributor to variations in AA digestibility. Maillard reactions are supported by high sugar concentrations, and significantly negative correlations between sugar concentration and the digestibility of 7 AA were observed in the present study.

There is no indication of the influence of tannins and InsP_6_ concentration on AA digestibility, although negative correlations between AA digestibility and tannins (Ortiz et al., 1993; Zuber et al., 2019) or InsP_6_ concentrations (Siegert et al., 2022a) were found in other feed ingredients. Such negative correlations are usually explained by AA-tannin complexes (Artz et al., 1987) or AA-InsP_6_ complexes (Selle et al., 2012), which reduce the efficacy of digestive enzymes. In the current study, however, the few significant correlations between AA digestibility and tannins or InsP_6_ are difficult to explain causatively because they were positive. It is likely that the effects of tannins and InsP_6_ on variations in AA digestibility were negligible, because tannin concentrations were low and the variation in InsP_6_ concentration was overall small.

The in vitro N solubility was unsuitable for predicting AA digestibility in the present study. This was supported by the negative correlations between in vitro N solubility and AA digestibility, as correlations should be positive to be causatively explicable. Possible reasons include a very low variation in in vitro N solubility among SBM samples. Lack of significant correlations between AA digestibility of SBM samples and the same in vitro approach were also reported for broiler chickens (Frikha et al., 2012).

For MEn, the determined positive and negative correlations with gross energy and crude fiber are likely due to the low energy content in the fiber fractions compared to most other analytical fractions. Negative correlations between crude fiber and ME in SBM samples were also described for broiler chickens (Ravindran et al., 2014).

### Predictions

We also calculated multiple linear regressions aiming to predict AA digestibility and MEn based on variables analyzed in the SBM samples, as performed previously (Siegert et al., 2022b). The predictive quality of these multiple regressions was neither consistent among the AA nor sufficiently accurate in most cases, as indicated by the adjusted R² and the root MS error of the relationships between the cross-validated predictions and observed values.

## CONCLUSIONS

Variations in the digestibility of SBM in cecectomized laying hens differed between AA, with the range in digestibility of 6 to 23%-units among the first-limiting AA Met, Cys, Thr, Tyr, and Val. The MEn varied considerably by 3.0 MJ/kg DM among the SBM samples. Our results provide no evidence of systematic deviations in AA digestibility or MEn of SBM samples between geographical regions. Common indicators of SBM quality and in vitro N solubility were not consistently related to AA digestibility and MEn. Further research is necessary to allow for rapid prediction of AA digestibility and MEn in SBM and other processing products of soybeans.

## Appendix Supplementary materials

Image, application 1

## Footnotes

Supplementary material associated with this article can be found in the online version at doi:10.1016/j.psj.2023.102580.
