# Supplementary material for: Amino acid digestibility and metabolizable energy of soybean meal of different origins in cecectomized laying hens
Source: Poult Sci. 2023 Feb 14;102(5):102580. doi: 10.1016/j.psj.2023.102580 (PMC10024217; doi:10.1016/j.psj.2023.102580)
Supplement: Supplementary file 1 [file mmc1.docx]

**Amino acid digestibility and metabolizable energy of soybean meal of different origins in cecectomised laying hens**

**Supplemental file**

**Table of contents**

[Supplemental Table 1. Concentrations of analyzed compounds in soybean meal variants](#SBManalysis)  2

[Supplemental Table 2. Concentrations of analyzed compounds in the experimental diets](#DietAnalysis)  5

Supplemental Table 3. Amino acid digestibility and MEn of the experimental diets 6

[Supplemental Table 4. Significant correlations between amino acid digestibility or MEn and
analyzed variables in soybean meal](#Correl)  7

[Supplemental Figure 1. Digestibility of selected first-limiting amino acids and MEn of soybean
meal from different geographical origins 9](#GeographicalOrigin)

[Supplemental Figure 2. Digestibility of selected first-limiting amino acids and MEn of not
genetically modified and genetically modified soybean meal variants](#GMO)  10

**Supplemental Table** **1.** Concentrations of analyzed compounds in soybean meal (g/kg DM, unless otherwise stated).

| Variant no.^1^ | 1 | 2 | 3 | 4 | 5 | 6 | 7 | 8 | 9 | 10 | 11 | 12 | 13 | 14 | 15 | 16 | 17 | 18 |
| --- | --- | --- | --- | --- | --- | --- | --- | --- | --- | --- | --- | --- | --- | --- | --- | --- | --- | --- |
| GMO^2^ | no | no | no | no | no | no | no | no | yes | yes | yes | yes | no | no | yes | yes | yes | yes |
| DM (g/kg) | 893 | 895 | 912 | 895 | 893 | 890 | 891 | 887 | 892 | 889 | 896 | 893 | 890 | 886 | 885 | 883 | 886 | 887 |
| CP | 516 | 534 | 485 | 515 | 533 | 522 | 515 | 543 | 532 | 506 | 564 | 563 | 556 | 549 | 511 | 498 | 503 | 499 |
| Ether extract | 20 | 21 | 30 | 25 | 21 | 15 | 21 | 19 | 24 | 32 | 26 | 21 | 17 | 20 | 28 | 29 | 27 | 22 |
| Crude ash | 70 | 82 | 75 | 76 | 71 | 70 | 72 | 79 | 72 | 73 | 73 | 76 | 73 | 78 | 83 | 100 | 81 | 76 |
| Crude fiber | 47 | 30 | 71 | 44 | 37 | 34 | 52 | 54 | 40 | 53 | 42 | 48 | 47 | 44 | 49 | 43 | 39 | 41 |
| aNDF_om_^3^ | 181 | 143 | 192 | 213 | 134 | 110 | 162 | 166 | 154 | 174 | 232 | 191 | 140 | 128 | 129 | 112 | 181 | 173 |
| ADF_om_^4^ | 82 | 55 | 102 | 88 | 71 | 67 | 84 | 80 | 66 | 86 | 63 | 62 | 79 | 80 | 65 | 59 | 56 | 56 |
| NDF-N^5^ | 5.2 | 8.6 | 4.5 | 12.3 | 6.5 | 3.7 | 7.4 | 4.2 | 7.6 | 5.8 | 20.3 | 12.5 | 5.9 | 5.5 | 4.5 | 3.1 | 7.8 | 10.0 |
| ADF-N^6^ | 1.1 | 0.7 | 0.8 | 1.0 | 0.8 | 0.9 | 1.0 | 0.9 | 0.9 | 0.9 | 1.6 | 1.4 | 1.1 | 1.1 | 0.8 | 0.7 | 1.0 | 1.2 |
| Starch | 62 | 59 | 53 | 63 | 55 | 64 | 57 | 48 | 54 | 52 | 46 | 47 | 53 | 51 | 62 | 68 | 70 | 70 |
| Sugar | 12 | 12 | 10 | 11 | 12 | 13 | 11 | 8 | 10 | 10 | 10 | 10 | 9 | 10 | 12 | 12 | 13 | 13 |
| Gross energy (MJ/kg DM) | 21.1 | 21.0 | 19.5 | 21.2 | 21.1 | 20.8 | 20.9 | 19.5 | 21.2 | 19.7 | 20.6 | 20.7 | 21.2 | 21.2 | 20.4 | 20.7 | 21.0 | 20.1 |
| Amino acids (g/16 g N) |  |  |  |  |  |  |  |  |  |  |  |  |  |  |  |  |  |  |
| Ala | 4.6 | 4.5 | 4.6 | 4.7 | 4.6 | 4.5 | 4.7 | 4.6 | 4.7 | 4.7 | 4.3 | 4.4 | 4.6 | 5.2 | 4.5 | 4.7 | 4.5 | 4.6 |
| Arg | 7.1 | 7.0 | 7.9 | 7.2 | 7.0 | 7.0 | 7.1 | 7.8 | 7.2 | 7.7 | 7.0 | 7.2 | 6.9 | 8.5 | 7.3 | 7.5 | 7.3 | 7.3 |
| Asx | 11.9 | 11.8 | 12.3 | 12.1 | 12.1 | 11.7 | 12.2 | 12.5 | 12.2 | 12.4 | 11.7 | 11.9 | 11.9 | 13.8 | 11.8 | 12.1 | 11.7 | 11.9 |
| Cys | 1.4 | 1.4 | 1.4 | 1.4 | 1.4 | 1.4 | 1.4 | 1.4 | 1.4 | 1.4 | 1.3 | 1.4 | 1.4 | 1.5 | 1.4 | 1.5 | 1.3 | 1.3 |
| Glx | 19.9 | 19.7 | 19.2 | 20.4 | 20.0 | 19.6 | 20.2 | 19.7 | 20.2 | 19.3 | 18.3 | 18.5 | 19.9 | 21.5 | 18.6 | 19.0 | 18.3 | 18.5 |
| Gly | 4.3 | 4.2 | 4.5 | 4.4 | 4.4 | 4.3 | 4.4 | 4.5 | 4.5 | 4.5 | 4.2 | 4.2 | 4.3 | 5.0 | 4.3 | 4.4 | 4.3 | 4.4 |
| His | 2.9 | 2.9 | 3.0 | 3.0 | 3.0 | 3.0 | 3.0 | 3.0 | 3.0 | 2.9 | 2.7 | 2.8 | 2.9 | 3.3 | 2.9 | 2.8 | 2.9 | 3.0 |
| Ile | 5.2 | 5.1 | 4.8 | 5.3 | 5.1 | 4.9 | 5.2 | 4.9 | 5.3 | 5.0 | 4.8 | 4.8 | 5.2 | 5.5 | 4.6 | 4.8 | 4.7 | 4.7 |
| Leu | 8.1 | 8.0 | 8.3 | 8.3 | 8.0 | 8.0 | 8.2 | 8.5 | 8.3 | 8.4 | 7.9 | 8.0 | 8.1 | 9.3 | 7.9 | 8.1 | 7.9 | 8.1 |
| Lys | 6.4 | 6.3 | 6.5 | 6.3 | 6.4 | 6.4 | 6.4 | 6.6 | 6.5 | 6.5 | 5.8 | 6.0 | 6.3 | 7.2 | 6.3 | 6.4 | 6.1 | 6.2 |
| Met | 1.5 | 1.5 | 1.6 | 1.5 | 1.5 | 1.5 | 1.5 | 1.6 | 1.5 | 1.5 | 1.4 | 1.4 | 1.4 | 1.6 | 1.5 | 1.5 | 1.4 | 1.5 |
| Phe | 5.9 | 5.8 | 5.3 | 6.0 | 5.8 | 5.8 | 6.1 | 5.5 | 6.1 | 5.5 | 5.3 | 5.3 | 6.0 | 6.3 | 5.1 | 5.3 | 5.2 | 5.3 |
| Pro | 5.0 | 5.0 | 5.2 | 5.2 | 5.0 | 4.9 | 5.1 | 5.3 | 5.2 | 5.2 | 5.2 | 5.3 | 5.1 | 6.0 | 5.3 | 5.1 | 5.1 | 5.3 |
| Ser | 5.0 | 5.0 | 5.6 | 5.1 | 5.0 | 5.1 | 5.2 | 5.6 | 5.1 | 5.5 | 5.2 | 5.2 | 5.0 | 6.2 | 5.3 | 5.3 | 5.2 | 5.4 |
| Thr | 4.2 | 4.2 | 4.1 | 4.3 | 4.3 | 4.3 | 4.3 | 4.1 | 4.3 | 4.1 | 3.9 | 3.9 | 4.2 | 4.6 | 4.0 | 4.1 | 4.0 | 4.1 |
| Tyr | 3.5 | 3.4 | 3.5 | 3.6 | 3.5 | 3.4 | 3.6 | 3.5 | 3.6 | 3.6 | 3.3 | 3.4 | 3.5 | 4.0 | 3.3 | 3.4 | 3.4 | 3.4 |
| Val | 4.8 | 4.7 | 4.9 | 5.0 | 4.7 | 4.6 | 4.8 | 5.0 | 4.9 | 5.1 | 4.8 | 4.9 | 4.8 | 5.6 | 4.8 | 5.0 | 4.9 | 5.0 |

Table continued on next page.

**Supplemental Table 1.** Continuation.

| Variant no.^1^ | 1 | 2 | 3 | 4 | 5 | 6 | 7 | 8 | 9 | 10 | 11 | 12 | 13 | 14 | 15 | 16 | 17 | 18 |
| --- | --- | --- | --- | --- | --- | --- | --- | --- | --- | --- | --- | --- | --- | --- | --- | --- | --- | --- |
| GMO^2^ | no | no | no | no | no | no | no | no | yes | yes | yes | yes | no | no | yes | yes | yes | yes |
| Inositol phosphate isomers (µmol/g DM)^7^ | | | | |  |  |  |  |  |  |  |  |  |  |  |  |  |  |
| InsP_6_ | 25.4 | 25.5 | 22.4 | 21.8 | 23.3 | 24.6 | 22.9 | 20.8 | 21.3 | 18.7 | 18.6 | 20.8 | 23.3 | 22.0 | 23.4 | 21.7 | 21.8 | 20.7 |
| Ins(1,2,3,4,6)P_5_ | 0.5 | 0.9 | 0.4 | 07 | 0.5 | 0.4 | 0.6 | 0.7 | 0.5 | 0.4 | 1.0 | 0.8 | 0.5 | 0.4 | ND | ND | 0.5 | 0.6 |
| Ins(1,2,3,4,5)P_5_ | 1.3 | 1.9 | 0.9 | 1.5 | 1.1 | 1.1 | 1.3 | 1.4 | 1.2 | 1.0 | 1.9 | 1.7 | 1.2 | 1.0 | 0.8 | 0.7 | 1.1 | 1.4 |
| Ins(1,2,4,5,6)P_5_ | 2.6 | 4.0 | 1.7 | 3.0 | 2.1 | 2.2 | 2.7 | 2.8 | 2.3 | 1.9 | 4.0 | 3.5 | 2.8 | 2.3 | 1.4 | 1.2 | 2.2 | 2.7 |
| Ins(1,2,5,6)P_4_ | ND | 0.5 | ND | 0.4 | ND | ND | 0.3 | 0.3 | 0.3 | ND | 0.8 | 0.6 | 0.3 | ND | ND | ND | ND | 0.4 |
| Ins(1,2,3,4)P_4_ | LOQ | 0.2 | LOQ | ND | LOQ | LOQ | LOQ | LOQ | LOQ | LOQ | 0.3 | ND | ND | LOQ | LOQ | LOQ | LOQ | LOQ |
| Tannins |  |  |  |  |  |  |  |  |  |  |  |  |  |  |  |  |  |  |
| Total phenols | 0.27 | 0.22 | 0.27 | 0.25 | 0.28 | 0.36 | 0.30 | 0.29 | 0.23 | 0.24 | 0.17 | 0.25 | 0.25 | 0.25 | 0.24 | 0.24 | 0.31 | 0.28 |
| Non-tannin phenols | 0.27 | 0.26 | 0.26 | 0.25 | 0.28 | 0.36 | 0.30 | 0.23 | 0.29 | 0.27 | 0.19 | 0.20 | 0.28 | 0.29 | 0.26 | 0.25 | 0.29 | 0.27 |
| Condensed tannins | <0.05 | <0.05 | <0.05 | <0.05 | <0.05 | <0.05 | <0.05 | <0.05 | <0.05 | <0.05 | <0.05 | <0.05 | <0.05 | <0.05 | <0.05 | <0.05 | <0.05 | <0.05 |
| Trypsin inhibitor activity | 4.54 | 2.70 | 1.62 | 1.06 | 2.90 | 4.34 | 2.97 | 3.32 | 2.95 | 2.58 | 0.79 | 1.93 | 3.98 | 4.52 | 3.17 | 4.00 | 1.90 | 2.22 |
| Urease activity (mg N/g/min) | <0.05 | <0.05 | <0.05 | <0.05 | <0.05 | <0.05 | <0.05 | <0.05 | <0.05 | <0.05 | <0.05 | <0.05 | <0.05 | <0.05 | <0.05 | <0.05 | <0.05 | <0.05 |
| KOH solubility (%) | 61.0 | 60.4 | 50.9 | 46.5 | 59.8 | 65.6 | 61.8 | 71.0 | 58.1 | 59.8 | 35.7 | 49.4 | 59.9 | 60.5 | 56.0 | 60.5 | 58.2 | 52.4 |
| Protein dispersibility  index (%) | 15.2 | 10.7 | 9.2 | 6.7 | 14.4 | 20.3 | 12.0 | 17.2 | 13.0 | 13.1 | 6.1 | 6.2 | 12.5 | 14.1 | 16.8 | 17.3 | 15.3 | 13.6 |
| *In vitro* N solubility (%) | 95 | 94 | 95 | 94 | 95 | 95 | 92 | 93 | 93 | 93 | 93 | 93 | 93 | 92 | 94 | 95 | 94 | 96 |
| Particle size distribution (%) | |  |  |  |  |  |  |  |  |  |  |  |  |  |  |  |  |  |
| 6.3–10 mm^8^ | 0 | 0 | 0 | 0 | 0 | 0 | 0 | 0 | 0 | 5.2 | 0 | 0 | 0 | 0 | 0 | 0 | 0 | 0 |
| 4.0–6.3 mm | 0 | 0 | 0 | 2.7 | 0 | 0 | 0.9 | 0 | 0 | 9.3 | 1.1 | 5.5 | 0 | 0 | 1.0 | 0 | 0 | 0 |
| 2.0–4.0 mm | 0.8 | 2.9 | 0.8 | 8.9 | 0.4 | 3.3 | 9.7 | 3.4 | 1.0 | 4.7 | 7.7 | 19.0 | 2.7 | 3.4 | 4.9 | 5.2 | 3.3 | 1.3 |
| 1.18–2.0 mm | 33.4 | 20.0 | 20.6 | 19.4 | 13.7 | 19.6 | 21.8 | 16.4 | 6.3 | 13.0 | 18.5 | 22.9 | 19.2 | 18.8 | 32.3 | 30.5 | 19.6 | 13.9 |
| 1.0–1.18 mm | 26.5 | 24.4 | 25.5 | 28.3 | 29.6 | 33.7 | 26.5 | 23.2 | 18.1 | 21.1 | 24.9 | 22.8 | 34.9 | 35.7 | 41.0 | 34.4 | 33.7 | 26.8 |
| 0.5–1.0 mm | 7.5 | 8.4 | 8.5 | 9.7 | 10.2 | 9.8 | 8.2 | 8.0 | 8.9 | 8.5 | 9.2 | 7.1 | 10.4 | 10.5 | 7.9 | 7.6 | 9.8 | 11.6 |
| 0.25–0.5 mm | 23.9 | 28.2 | 25.5 | 24.7 | 31.1 | 25.3 | 24.6 | 29.7 | 36.9 | 27.9 | 29.8 | 18.3 | 25.9 | 26.3 | 12.1 | 16.2 | 25.3 | 39.6 |
| 0.125–0.25 mm | 6.8 | 11.1 | 13.0 | 6.0 | 10.7 | 6.3 | 6.7 | 12.9 | 20.4 | 8.4 | 7.8 | 3.9 | 6.3 | 4.7 | 0.7 | 3.8 | 6.3 | 6.6 |
| 0.063–0.125 mm | 1.0 | 3.6 | 4.8 | 1.0 | 3.4 | 1.5 | 1.4 | 4.7 | 6.7 | 1.6 | 1.0 | 0.5 | 0.5 | 0.6 | 0 | 1.1 | 1.5 | 0.1 |
| <0.063 mm | 0.2 | 1.3 | 1.2 | 0.2 | 0.9 | 0.5 | 0.2 | 1.6 | 1.7 | 0.4 | 0.1 | 0 | 0 | 0.1 | 0.3 | 1.2 | 0.5 | 0.2 |

Table continued on next page.

**Supplemental Table 1.** Continuation.

| Variant no.^1^ | 1 | 2 | 3 | 4 | 5 | 6 | 7 | 8 | 9 | 10 | 11 | 12 | 13 | 14 | 15 | 16 | 17 | 18 |
| --- | --- | --- | --- | --- | --- | --- | --- | --- | --- | --- | --- | --- | --- | --- | --- | --- | --- | --- |
| GMO^2^ | no | no | no | no | no | no | no | no | yes | yes | yes | yes | no | no | yes | yes | yes | yes |
| Regression of particle size distribution^9^ (SE of estimates in parentheses) | | | | | | | | |  |  |  |  |  |  |  |  |  |  |
| a (rate constant) | 2.95  (0.28) | 2.85  (0.22) | 2.85  (0.23) | 2.81  (0.25) | 3.24  (0.28) | 3.17  (0.26) | 2.74  (0.23) | 2.89  (0.24) | 5.49  (2.02) | 2.43  (0.31) | 2.90  (0.25) | 2.16  (0.18) | 3.32  (0.30) | 3.37  (0.36) | 9.8  (1.18) | 3.18  (0.34) | 3.49  (0.35) | 3.51  (0.27) |
| b (inflection point) | 1.01  (0.04) | 0.82  (0.04) | 0.80  (0.04) | 1.01  (0.04) | 0.76  (0.04) | 0.93  (0.03) | 1.01  (0.03) | 0.76  (0.04) | 0.45  (0.06) | 0.98  (0.06) | 0.92  (0.04) | 1.30  (0.05) | 0.93  (0.03) | 0.94  (0.04) | 1.13  (<0.01) | 1.10  (0.03) | 0.58  (0.04) | 0.77  (0.03) |
| R² | 0.97 | 0.97 | 0.97 | 0.97 | 0.96 | 0.98 | 0.98 | 0.96 | 0.96 | 0.95 | 0.97 | 0.98 | 0.97 | 0.96 | >0.99 | 0.98 | 0.96 | 0.96 |
| Root MS error | 7.4 | 7.1 | 7.4 | 6.7 | 8.1 | 6.7 | 6.5 | 7.6 | 7.7 | 8.7 | 7.3 | 5.9 | 7.4 | 8.4 | 4.2 | 6.4 | 7.9 | 8.3 |

^1^Country of origin of variant no.: 1 and 6 Ukraine; 2, 3, and 19 Germany; 4 France; 5 Italy; 7 Russia; 8 India; 9–14 Brazil; 15 United States; 16 ⅓ United States and ⅔ Canada; 17 and 18 Argentina.

^2^GMO = genetically modified.

^3^Neutral detergent fiber, determined without residual ash and after treatment with α-amylase.

^4^Acid detergent fiber, determined without residual ash.

^5^Neutral detergent insoluble nitrogen.

^6^Acid detergent insoluble nitrogen.

^7^LOQ = below limit of determination; ND = below limit of detection.

^8^No particles larger than 10 mm.

^9^Slope of the regression indicates homogeneity of particle size distribution (the higher the more heterogenous); inflection points indicate the average particle size.

**Supplemental Tab****le 2.** Concentrations of analyzed compounds in the experimental diets (g/kg DM, unless otherwise stated).

| Diet^1^ | BD | 1 | 2 | 3 | 4 | 5 | 6 | 7 | 8 | 9 | 10 | 11 | 12 | 13 | 14 | 15 | 16 | 17 | 18 |
| --- | --- | --- | --- | --- | --- | --- | --- | --- | --- | --- | --- | --- | --- | --- | --- | --- | --- | --- | --- |
| DM (g/kg) | 944 | 925 | 937 | 974 | 932 | 932 | 934 | 936 | 964 | 933 | 932 | 938 | 954 | 932 | 997 | 927 | 931 | 929 | 935 |
| CP | 196 | 351 | 358 | 325 | 355 | 360 | 351 | 353 | 348 | 359 | 351 | 365 | 361 | 358 | 350 | 346 | 343 | 346 | 343 |
| Trypsin inhibitor activity | 0.69 | 1.60 | 1.40 | 1.01 | 1.09 | 0.99 | 1.45 | 1.42 | 1.67 | 0.90 | 1.22 | 0.79 | 1.12 | 1.37 | 1.75 | 1.06 | 1.09 | 0.99 | 1.02 |
| Gross energy (MJ/kg DM) | 18.0 | 18.9 | 19.0 | 18.0 | 19.1 | 19.0 | 18.8 | 19.0 | 18.2 | 19.0 | 18.9 | 18.9 | 18.7 | 18.7 | 18.4 | 18.9 | 18.8 | 18.9 | 18.6 |
| Amino acids |  |  |  |  |  |  |  |  |  |  |  |  |  |  |  |  |  |  |  |
| Ala | 8.7 | 15.9 | 15.9 | 15.4 | 14.7 | 15.5 | 16.4 | 16.9 | 16.6 | 16.8 | 15.7 | 15.6 | 15.9 | 16.0 | 14.5 | 14.9 | 14.3 | 14.7 | 14.7 |
| Arg | 10.2 | 20.9 | 21.0 | 21.6 | 19.3 | 20.4 | 22.3 | 22.9 | 23.5 | 22.6 | 21.7 | 21.7 | 22.5 | 20.7 | 20.4 | 20.6 | 19.8 | 20.4 | 20.2 |
| Asx | 15.1 | 33.7 | 34.0 | 32.4 | 31.1 | 33.0 | 34.7 | 35.7 | 35.8 | 35.4 | 33.3 | 34.2 | 35.9 | 33.9 | 31.4 | 31.6 | 30.3 | 31.2 | 31.2 |
| Cys | 3.5 | 5.3 | 5.4 | 5.4 | 5.0 | 5.3 | 5.4 | 5.7 | 5.7 | 5.5 | 5.4 | 5.4 | 5.1 | 5.4 | 4.9 | 5.0 | 4.8 | 5.0 | 4.9 |
| Glx | 53.9 | 85.2 | 85.4 | 79.0 | 81.4 | 85.4 | 86.2 | 89.5 | 85.2 | 87.2 | 79.8 | 80.7 | 81.6 | 88.4 | 76.5 | 77.2 | 74.8 | 76.6 | 75.8 |
| Gly | 7.8 | 14.5 | 14.5 | 14.3 | 13.5 | 14.2 | 15.2 | 15.8 | 15.2 | 15.4 | 14.4 | 14.7 | 14.9 | 14.7 | 13.5 | 13.7 | 13.3 | 13.7 | 13.7 |
| His | 5.0 | 9.7 | 9.7 | 9.2 | 9.1 | 9.4 | 10.1 | 10.2 | 9.9 | 10.2 | 9.3 | 9.4 | 9.5 | 9.7 | 8.8 | 8.8 | 8.6 | 9.0 | 8.9 |
| Ile | 8.1 | 17.0 | 17.2 | 15.3 | 15.8 | 16.8 | 17.0 | 17.4 | 16.7 | 17.0 | 15.4 | 15.8 | 16.0 | 17.3 | 14.6 | 14.6 | 14.2 | 14.7 | 14.8 |
| Leu | 16.7 | 29.2 | 29.2 | 28.3 | 27.4 | 28.6 | 30.2 | 31.1 | 30.7 | 30.7 | 28.6 | 29.2 | 29.9 | 29.6 | 27.1 | 27.4 | 26.5 | 27.1 | 27.2 |
| Lys | 9.0 | 19.1 | 19.3 | 18.0 | 17.6 | 18.7 | 20.3 | 20.3 | 19.5 | 20.1 | 18.3 | 18.1 | 19.3 | 19.2 | 17.2 | 17.4 | 17.0 | 17.2 | 17.2 |
| Met | 3.7 | 5.9 | 6.0 | 5.9 | 5.6 | 6.0 | 6.3 | 6.6 | 6.4 | 6.2 | 5.8 | 5.8 | 6.2 | 6.0 | 5.4 | 5.7 | 5.4 | 5.5 | 5.5 |
| Phe | 10.5 | 20.8 | 20.9 | 18.0 | 19.5 | 20.5 | 20.1 | 21.0 | 19.6 | 20.6 | 18.5 | 18.8 | 19.1 | 21.4 | 17.5 | 17.4 | 16.9 | 17.3 | 17.3 |
| Pro | 18.0 | 24.5 | 24.9 | 24.7 | 23.7 | 24.8 | 26.2 | 27.6 | 25.9 | 26.3 | 24.9 | 26.3 | 24.3 | 25.2 | 24.8 | 24.3 | 24.1 | 24.7 | 25.0 |
| Ser | 10.1 | 17.1 | 17.2 | 17.8 | 16.2 | 16.8 | 18.5 | 19.2 | 19.5 | 19.0 | 18.1 | 18.5 | 19.2 | 17.6 | 17.2 | 17.2 | 16.6 | 16.9 | 16.8 |
| Thr | 6.7 | 13.6 | 13.7 | 12.7 | 12.8 | 13.4 | 13.7 | 14.1 | 13.9 | 14.0 | 12.8 | 13.0 | 13.4 | 13.8 | 12.1 | 12.2 | 11.8 | 12.2 | 12.2 |
| Tyr | 6.3 | 12.0 | 12.1 | 11.5 | 11.2 | 11.8 | 12.5 | 12.9 | 12.5 | 12.7 | 11.3 | 11.7 | 12.0 | 12.3 | 10.9 | 10.9 | 10.7 | 11.0 | 11.0 |
| Val | 9.2 | 16.3 | 16.5 | 16.5 | 15.2 | 16.1 | 17.5 | 18.1 | 17.8 | 17.6 | 16.6 | 16.6 | 16.7 | 16.4 | 15.5 | 15.8 | 15.3 | 15.8 | 15.9 |

^1^BD = basal diet and diets containing the solvent-extracted soybean meal variants.

**Supplemental Table** **3.** Amino acid digestibility (%) and MEn (MJ/kg DM) of the experimental diets (arithmetic means).

| Diet | Ala | Arg | Asx | Cys | Glx | His | Ile | Leu | Lys | Met | Phe | Pro | Ser | Thr | Tyr | Val | MEn |
| --- | --- | --- | --- | --- | --- | --- | --- | --- | --- | --- | --- | --- | --- | --- | --- | --- | --- |
| BD | 79.9 | 89.3 | 77.9 | 78.9 | 93.2 | 82.4 | 87.3 | 88.9 | 82.5 | 89.6 | 90.4 | 91.7 | 82.6 | 72.3 | 86.9 | 83.8 | 14.2 |
| 1 | 79.3 | 90.6 | 80.7 | 75.9 | 91.6 | 84.2 | 88.0 | 87.1 | 85.9 | 88.9 | 90.0 | 88.6 | 82.5 | 77.4 | 88.1 | 83.8 | 11.9 |
| 2 | 81.4 | 91.3 | 81.6 | 78.4 | 92.3 | 85.4 | 89.5 | 88.4 | 86.5 | 89.3 | 91.2 | 90.3 | 83.6 | 78.6 | 88.8 | 85.1 | 12.3 |
| 3 | 81.5 | 92.0 | 81.7 | 79.4 | 92.0 | 84.4 | 88.4 | 88.4 | 86.2 | 90.1 | 89.9 | 90.2 | 84.5 | 78.3 | 87.9 | 86.3 | 11.3 |
| 4 | 81.2 | 91.0 | 79.7 | 76.0 | 91.9 | 83.7 | 88.8 | 88.1 | 84.6 | 89.9 | 90.7 | 89.9 | 82.8 | 77.5 | 88.2 | 84.5 | 12.2 |
| 5 | 81.5 | 91.4 | 81.7 | 78.1 | 92.5 | 84.6 | 89.3 | 88.4 | 86.6 | 90.6 | 91.3 | 90.5 | 83.4 | 78.3 | 89.0 | 85.1 | 12.1 |
| 6 | 81.8 | 91.8 | 81.9 | 77.0 | 92.0 | 85.1 | 88.8 | 88.2 | 87.2 | 89.8 | 90.2 | 90.5 | 84.1 | 78.3 | 89.2 | 85.8 | 12.0 |
| 7 | 82.9 | 92.1 | 82.9 | 79.1 | 92.6 | 85.7 | 89.5 | 89.2 | 86.9 | 90.4 | 91.1 | 90.8 | 85.4 | 79.6 | 89.8 | 86.9 | 12.1 |
| 8 | 85.1 | 93.5 | 86.7 | 83.2 | 93.5 | 88.0 | 90.7 | 90.5 | 89.3 | 91.8 | 91.7 | 92.0 | 88.3 | 83.4 | 90.7 | 88.9 | 11.5 |
| 9 | 83.5 | 92.1 | 82.6 | 78.9 | 92.6 | 85.6 | 89.4 | 89.1 | 87.3 | 90.8 | 91.2 | 90.9 | 85.3 | 79.6 | 89.7 | 86.5 | 12.3 |
| 10 | 80.7 | 91.9 | 82.1 | 79.6 | 91.9 | 84.7 | 88.2 | 88.2 | 85.8 | 90.3 | 89.9 | 90.8 | 84.7 | 78.0 | 87.8 | 85.7 | 12.2 |
| 11 | 80.7 | 91.2 | 78.4 | 76.6 | 90.3 | 82.3 | 87.9 | 88.0 | 83.6 | 89.1 | 89.7 | 89.8 | 83.6 | 76.6 | 87.8 | 85.1 | 12.1 |
| 12 | 81.4 | 91.9 | 80.5 | 76.0 | 91.3 | 84.0 | 88.5 | 88.6 | 85.5 | 90.6 | 90.3 | 89.7 | 84.5 | 77.6 | 88.3 | 85.6 | 11.9 |
| 13 | 82.4 | 91.5 | 81.8 | 78.9 | 92.7 | 84.7 | 89.6 | 88.7 | 86.8 | 90.7 | 91.4 | 90.5 | 84.4 | 79.5 | 89.4 | 85.7 | 12.1 |
| 14 | 79.4 | 91.1 | 80.4 | 77.5 | 91.4 | 84.0 | 87.2 | 87.1 | 85.1 | 88.7 | 89.1 | 90.1 | 83.5 | 75.8 | 87.3 | 84.3 | 11.8 |
| 15 | 82.0 | 92.1 | 82.8 | 79.6 | 92.4 | 85.0 | 89.0 | 88.9 | 86.7 | 90.8 | 90.4 | 91.3 | 85.3 | 78.3 | 88.5 | 86.5 | 12.2 |
| 16 | 79.4 | 90.6 | 80.8 | 75.6 | 91.0 | 83.7 | 87.1 | 86.8 | 85.6 | 88.9 | 88.6 | 89.8 | 82.8 | 75.9 | 86.9 | 84.4 | 11.9 |
| 17 | 79.7 | 91.0 | 80.5 | 76.9 | 91.3 | 83.2 | 87.6 | 87.3 | 84.7 | 89.1 | 89.2 | 89.8 | 83.2 | 76.1 | 87.3 | 84.8 | 12.0 |
| 18 | 78.1 | 90.6 | 78.6 | 74.1 | 90.6 | 82.4 | 87.1 | 86.8 | 83.4 | 88.7 | 88.6 | 89.5 | 82.4 | 74.7 | 86.6 | 84.2 | 11.8 |
| 19 | 80.9 | 90.3 | 80.2 | 76.8 | 91.4 | 82.5 | 87.3 | 87.7 | 84.2 | 88.4 | 89.5 | 89.7 | 82.4 | 75.0 | 87.1 | 84.2 | 13.3 |
| Pooled SD | 3.7 | 1.5 | 2.9 | 4.6 | 1.5 | 2.5 | 2.3 | 2.3 | 2.2 | 2.6 | 2.0 | 2.0 | 2.7 | 3.7 | 2.1 | 2.7 | 0.42 |

^1^BD = basal diet and diets containing the solvent-extracted soybean meal variants.

**Supplemental Table 4****.** Significant (*P*<0.050) correlations between amino acid digestibility (%) or MEn (MJ/kg DM) and analyzed variables in solvent-extracted soybean meal (n = 18; g/kg dry matter unless otherwise stated).^1^

|  | Amino acid digestibility | | | | | | | | | | | | | | | |  |
| --- | --- | --- | --- | --- | --- | --- | --- | --- | --- | --- | --- | --- | --- | --- | --- | --- | --- |
|  | Ala | Arg | Asx | Cys | Glx | His | Ile | Leu | Lys | Met | Phe | Pro | Ser | Thr | Tyr | Val | MEn |
| Crude fiber |  | 0.56 |  |  |  |  |  |  |  | 0.47 |  |  | 0.55 |  |  | 0.53 | -0.65^**^ |
| aNDFom |  |  | -0.57 |  |  | -0.57 |  |  | -0.64^**^ |  |  | -0.54 |  |  |  |  |  |
| ADFom | 0.49 | 0.60^**^ |  | 0.61^**^ | 0.55 |  | 0.54 | 0.57 |  | 0.64^**^ | 0.52 |  | 0.48 | 0.62^**^ | 0.54 | 0.55 |  |
| NDF-N |  |  | -0.81^***^ |  | -0.58^*^ | -0.71^***^ |  |  | -0.77^***^ |  |  |  |  |  |  |  |  |
| ADF-N |  |  | -0.69^**^ |  | -0.55^*^ | -0.62^**^ |  |  | -0.67^**^ |  |  |  |  |  |  |  |  |
| Starch | -0.57 | -0.56 |  | -0.56 |  |  |  | -0.60 |  | -0.50 |  |  | -0.54 |  | -0.61^**^ | -0.60^**^ |  |
| Sugar | -0.48 | -0.52 |  | -0.53 |  |  |  | -0.51 |  |  |  |  | -0.56 |  | -0.51 | -0.57 |  |
| Gross energy (MJ/kg DM) |  |  |  |  |  |  |  |  |  |  |  |  |  |  |  |  | 0.59 |
| InsP_6_ |  |  |  |  | 0.58 | 0.53 | 0.52 |  | 0.59^**^ |  | 0.50 |  |  |  |  |  |  |
| Ins(1,2,3,4,5)P_5_ (µmol/g DM) |  |  | -0.51 |  |  |  |  |  |  |  |  |  |  |  |  |  |  |
| Ins(1,2,4,5,6)P_5_ (µmol/g DM) |  |  | -0.48 |  |  |  |  |  |  |  |  |  |  |  |  |  |  |
| Tannin phenols |  |  |  |  | 0.51 | 0.49 |  |  | 0.50 |  |  |  |  |  |  |  |  |
| Trypsin inhibitor activity (SBM) |  |  | 0.58 |  | 0.47 | 0.61^**^ |  |  | 0.67^**^ |  |  |  |  |  |  |  |  |
| Trypsin inhibitor activity (diet) |  |  |  |  |  |  |  |  |  |  |  | 0.55 | 0.49 |  |  |  |  |
| KOH solubility (%) |  |  | 0.78^***^ |  | 0.60^**^ | 0.71^***^ |  |  | 0.73^***^ |  |  |  |  |  |  |  |  |
| Protein dispersibility index (%) |  |  | 0.52 |  |  |  |  |  | 0.47 |  |  |  |  |  |  |  |  |
| *In vitro* N solubility (%) | -0.54 |  |  |  |  |  |  | -0.50 |  |  |  |  | -0.54 |  | -0.50 |  |  |
| Amino acids |  |  |  |  |  |  |  |  |  |  |  |  |  |  |  |  |  |
| Ala |  |  |  |  |  |  |  |  |  |  |  |  |  |  | 0.61^**^ |  |  |
| Arg |  |  |  |  |  |  |  |  |  |  |  |  |  |  | 0.54 | 0.61^**^ |  |
| Asx |  |  |  |  |  |  |  |  |  |  |  |  |  |  | 0.64^**^ | 0.54 |  |
| Cys |  |  |  |  |  |  |  |  |  |  |  |  |  |  | 0.53 |  |  |
| Glx | 0.51 |  |  |  |  |  | 0.52 |  |  |  | 0.61^**^ |  |  |  | 0.69^**^ |  |  |
| Gly |  |  |  |  |  |  |  |  |  |  |  |  |  |  | 0.63^**^ | 0.53 |  |
| His |  |  |  |  |  |  |  |  |  |  |  |  |  |  | 0.76^***^ | 0.61^**^ |  |
| Ile |  |  |  |  |  |  |  |  |  |  | 0.58 |  |  |  | 0.59^**^ |  |  |
| Leu |  |  |  |  |  |  |  |  |  |  |  |  |  |  | 0.64^**^ | 0.53 |  |
| Lys |  | 0.49 |  | 0.53 |  |  |  |  |  |  |  |  |  |  | 0.73^***^ | 0.60^**^ |  |
| Met |  | 0.49 |  | 0.52 |  |  |  |  |  |  |  |  |  |  | 0.73^***^ | 0.63^**^ |  |
| Phe | 0.53 |  |  |  | 0.48 |  | 0.55 |  |  |  | 0.66^**^ |  |  |  | 0.62^**^ |  |  |
| Pro |  |  |  |  |  |  |  |  |  |  |  |  |  |  | 0.47 |  |  |

Table continued on next page

**Supplemental Table 4.** Continuation.

|  | Amino acid digestibility | | | | | | | | | | | | | | | |  |
| --- | --- | --- | --- | --- | --- | --- | --- | --- | --- | --- | --- | --- | --- | --- | --- | --- | --- |
|  | Ala | Arg | Asx | Cys | Glx | His | Ile | Leu | Lys | Met | Phe | Pro | Ser | Thr | Tyr | Val | MEn |
| Amino acids |  |  |  |  |  |  |  |  |  |  |  |  |  |  |  |  |  |
| Ser |  |  |  |  |  |  |  |  |  |  |  |  |  |  | 0.54 | 0.61^**^ |  |
| Thr |  |  |  |  |  |  | 0.48 |  |  |  | 0.56 |  |  |  | 0.69^**^ |  |  |
| Tyr |  |  |  |  |  |  |  |  |  |  |  |  |  |  | 0.64^**^ |  |  |
| Amino acids (g/16 g N) |  |  |  |  |  |  |  |  |  |  |  |  |  |  |  |  |  |
| Arg |  |  |  |  |  |  |  |  |  |  |  |  |  |  |  | 0.59 | -0.56 |
| Asx |  | 0.51 |  | 0.49 |  |  |  |  |  |  |  |  |  |  | 0.61^**^ | 0.67^**^ |  |
| Cys |  |  |  |  |  | 0.54 |  |  | 0.47 |  |  |  |  |  |  |  |  |
| Glx | 0.49 |  |  | 0.51 | 0.67^**^ | 0.61^**^ | 0.61^**^ | 0.47 | 0.50 |  | 0.66^**^ |  |  | 0.49 | 0.70^**^ |  |  |
| Gly |  |  |  |  |  |  |  |  |  |  |  |  |  |  |  | 0.56 |  |
| His |  | 0.50 |  |  |  |  |  |  |  |  |  |  |  |  | 0.66^**^ | 0.65^**^ |  |
| Ile |  |  |  |  | 0.56 |  | 0.51 |  |  |  | 0.62^**^ |  |  |  | 0.58 |  |  |
| Leu |  | 0.48 |  |  |  |  |  |  |  |  |  |  |  |  | 0.58 | 0.64^**^ |  |
| Lys |  | 0.59 | 0.57 | 0.60^**^ | 0.50 | 0.66^**^ |  |  | 0.56 |  |  |  |  |  | 0.61^**^ | 0.63^**^ |  |
| Met |  | 0.61^**^ | 0.49 | 0.62^**^ |  | 0.53 |  |  |  |  |  |  |  |  | 0.59 | 0.68^**^ |  |
| Phe | 0.51 |  |  |  | 0.64^**^ | 0.51 | 0.61^**^ |  |  |  | 0.70^**^ |  |  |  | 0.60^**^ |  |  |
| Pro |  |  |  |  |  |  |  |  |  |  |  |  |  |  |  | 0.48 |  |
| Ser |  |  |  |  |  |  |  |  |  |  |  |  |  |  |  | 0.64^**^ | -0.53 |
| Thr |  |  |  |  | 0.59 | 0.56 |  |  |  |  | 0.49 |  |  |  | 0.59 |  |  |
| Tyr |  |  |  |  |  |  |  |  |  |  |  |  |  |  | 0.60^**^ | 0.54 |  |

^1**^*P*≤0.01; ^***^*P*≤0.001.

**Supplemental Figure 1.** Scatter-dot plots of the digestibility of selected first-limiting amino acids and MEn of solvent-extracted soybean meal from different geographical origins. Note that the ordinate has a different scale for each amino acid.

**Supplemental Figure 2.** Scatter-dot plots of the digestibility of selected first-limiting amino acids and MEn of not genetically modified (**nonGMO**) and genetically modified (**GMO**) solvent-extracted soybean meal variants. Argentinean soybean meal variants are marked in grey. Note that the ordinate has a different scale for each amino acid.
